# Whole exome sequencing revealed a pathogenic variant in a gene related to malignant hyperthermia in a Vietnamese cardiac surgical patient: A case report

**DOI:** 10.1016/j.amsu.2019.10.030

**Published:** 2019-11-06

**Authors:** Tran-Thuy Nguyen, Ngoc-Thanh Le, Thuy-Mau Thi Nguyen, Huy-Hoang Nguyen, Kim-Lien Thi Nguyen, Long Doan Dinh, The-Binh Nguyen, Anh Tien Do, Cong Huu Nguyen, Trung-Hieu Nguyen, Hong-Nhung Thi Pham, Thom Thi Vu

**Affiliations:** a Department of Cardiovascular and Thoracic Surgery, Cardiovascular Center, E Hospital, 87–89 Tran Cung, Cau Giay, Hanoi, Viet Nam; b School of Medicine and Pharmacy, Vietnam National University Hanoi, 144 Xuan Thuy Street, Cau Giay, Hanoi, Viet Nam; c Institute of Genome Research, Vietnam Academy of Science and Technology, 18 Hoang Quoc Viet, Cau Giay, Hanoi, Viet Nam; d Graduate University of Science and Technology, Vietnam Academy of Science and Technology, 18 Hoang Quoc Viet, Cau Giay, Hanoi, Viet Nam

**Keywords:** Anaesthetics, *CACNA1S*, Case report, Malignant hyperthermia, *RYR1*

## Abstract

**Introduction:**

Malignant hyperthermia (MH) is a rare autosomal dominant pharmacogenetic disorder which known associated with some genes such as *CACNA1S* and *RYR1*. Using whole exome analysis, we aimed to find out the genetic variant data in a malignant hyperthermia patient undergoing cardiac surgery.

**Presentation of case:**

Patient was 59 years old male with dull left chest pain, mild breathing difficulty, thrombosis in the left atrium, mitral valve stenosis that needed a surgery to remove the thrombus and replace the mitral valve. After 5-h operation of left mitral heart valve replacement using both intravenous and inhaled anaesthetics, the patient showed suddenly hyperthermia (39.5 °C), low blood pressure (90/50 mmHg), heavy sweating, 1 mm dilated pupils on both sides, positive light reflection. Whole exome analysis showed 96,286 of SNPs including 11,705 of synonymous variants, 11,388 of missense variants, 106 of stop gained, and 39 of stop lost. One variant of *RYR1* gene was found as mutation point at c.7048G > A (p.Ala2350Thr) known related to MH.

**Discussion:**

This was a rare case of MH during cardiac surgery reported in Vietnam that might related to mutation point at c.7048G > A (p.Ala2350Thr) of *RYR1* gene.

**Conclusion:**

Patient carried a mutant of *RYR1* gene could possibly lead to MH development post anaesthesia of cardiac surgery.

## Highlights

• A male cardiac surgery patient got malignant hyperthermia post anaesthesia.
• Whole exome variant analysis showed a mutant variant in *RYR1* gene of patient.
• *RYR1* gene testing might need to avoid MH during surgery using inhaled anaesthetics.

## 1 Introduction

Malignant hyperthermia (MH), a rare autosomal dominant pharmacogenetic disorder with proportion ranging from 1:10,000 to 1:250,000, is triggered in susceptible individuals to inhaled anaesthetic such as isoflurane, desflurane, sevoflurane or enflurane [[1], [2], [3]]. Clinical manifestations can vary according to different stages, with typical early signs including increased ETCO_2_, tachycardia, jaw muscle rigidity or body stiffness. In the late state, patients exhibit an increase in body temperature, rhabdomyolysis leading to arrhythmia and acute renal failure [2]. *RYR1* and *CACNA1S* genes which encodes a ryanodine receptor of the skeletal muscle Ca^2+^ release channel and the voltage-gated DHPR, respectively were counted for 50 to 70% of the MH case [4]. However, among more than 400 known *RYR1* variants, only 42 *RYR1* and 2 *CACNA1S* variants are confirmed as MH causative and can be used in diagnostic genetic testing for MH [5]. During last five years, scientists from all over the world reported less than 10 cases of MH related to trauma [6] or cardiac surgery [7]. In Vietnam, there have been very rare reports of MH associated with cardiac surgery. To date, this is the first case report of MH related to heart valve replacement surgery in response to inhaled anaesthetic drugs in Vietnam with clinical development and whole exome analysis. This work has been reported in line with SCARE criteria [8].

## 2 Presentation of case

### 2.1 Patient's paraclinical and clinical data

**Fig. 1:**
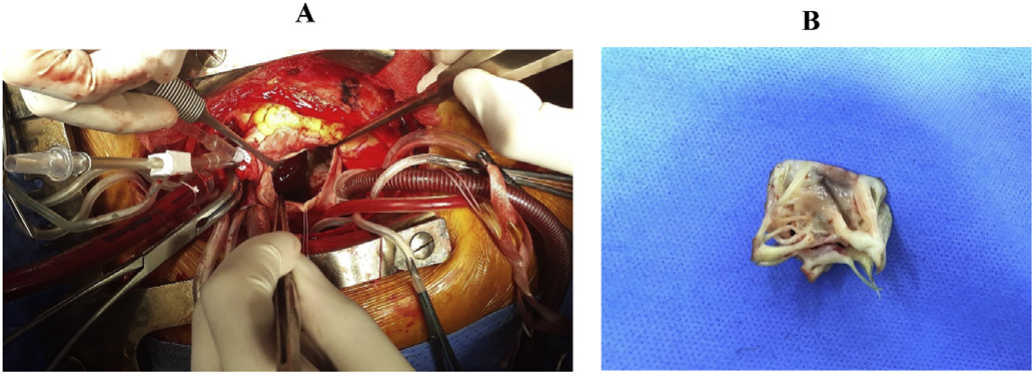
Heart valve replacement of the patient. A: The cardiac surgery position. B: The cut off mitral valve.

Patient was male, 59 years old, weight of 52 kg with a history of close mitral commissurotomy 22 years ago, hospitalized due to a dull left chest pain, class II of breath with mild shortness of breath classified by NYHA (2016) within one month. At the day of admission, patient showed consciousness, hemodynamic stability, normal heart rate, pulse 83 beats/minute, blood pressure 160/100 mmHg, breathing rate 18 times/minute, temperature 36 °C. The ultrasound result showed a thrombosis in the left atrium and mitral valve stenosis which needed a surgery as shown in Fig. 1.

The patient was given general anaesthesia including Esmeron 50 mg, Isofluran 250 mg and Sevoflurane 250 mg; maintaining anaesthesia with Diprivan (propofol) 100 mg–50 mg – 30 mg; Fentanyl 0.25 mg–0.2 mg – 0.1 mg. After 280 minutes of the surgery, the patient showed suddenly hyperthermia (39.5 °C), low blood pressure (90/50 mmHg), heavy sweating, 1 mm pinpoint pupils on both sides, positive pupillary light reflex and abnormal blood gases and electrolytes. We supposed that this was a case of MH. Therefore, the patient was treated in the direction of MH by controlled ventilation with FiO_2_ 60–100%, Cefuroxime 250 mg; Dobutamine 250 mg; Ephedrine 30 mg; Fentanyl 100 μg/hr, positive cooling with ice water until the patient's body temperature dropped; furosemide diuretic 20 mg (1 tube), Sodium bicarbonate 4.2% (2 bottles), Sodium Chloride 0.9% intravenous solution, establishing central venous line, non-invasive arterial blood pressure; coagulation test per 4 hours; blood gas test. Dantrolene was not available at this time for the patient therefore he was not treated with the specific treatment drug. At the later time points of surgery day, the patient showed signs of recovery. Two days later, patient needed the second operation due to post-operation bleeding of mitral valve replacement. One month later, the third surgery was performed for the patient for pericardial blood clot. Both surgeries were performed with intravenous anaesthetic instead of inhaled anaesthetic with Sevofluran so the patient did not show any symptoms of MH.

### 2.2 Patient's whole exome sequencing data

Suspected MH patient was drawn blood samples for whole exome analysis using next generation sequencing. More than 169 million 150 bp DNA reads were sequenced across the sample with 99.9% of reads were successfully mapped to the human genome, including 10,429,171,492 bases. Filtering on sequence quality and mapping score revealed the percentage of bases with quality scores (Qphred) higher than 20 or 30 (supplemental methods). We have identified 96,286 of SNPs including 11,705 of synonymous variants, 11,388 of missense variants, 106 of stop gained, and 39 of stop lost. A total 13,692 of INDEL were also detected including 321 of frameshift variants, 178 of inframe insertion, and 207 of inframe deletion. SNPs and indels were detected on the patient's coding genome at a Het/Hom rate of 1.3.

**Fig. 2:**
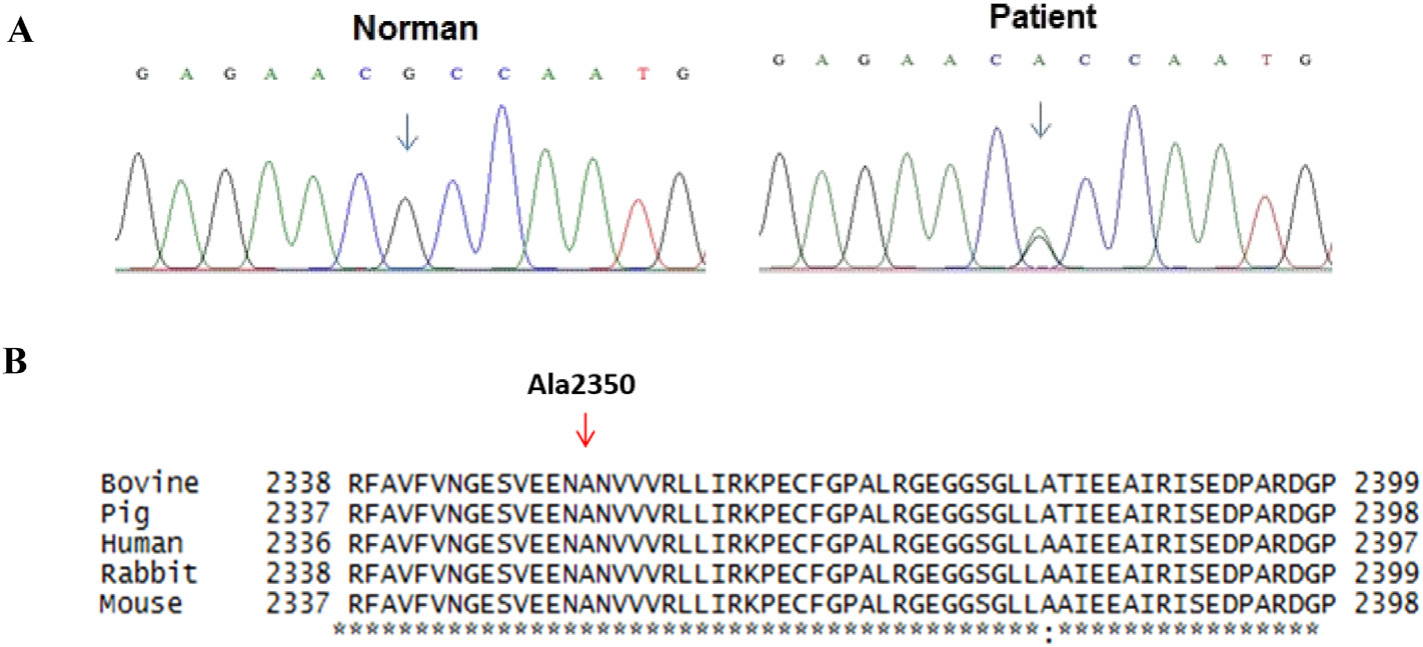
**A:** The mutation point (c.7048G > A, p.Ala2350Thr) in *RYR1* gene in patient that was confirmed by Sanger sequencing. **B**: Comparison the first-order structure of RYR1 protein molecular among species: human (XM011527205), bovine (NM001206777), pig (NM001001534), rabbit (NM001101718), and mouse (AY268935).

Targeting analysis for genes involved in the disease, we identified 15 SNPs on *CACNA1S* gene and 18 SNPs in *RYR1* gene including 7 synonymous variants and one missense mutation (codon 2350, c.7048G > A, p.Ala2350Thr, polyphen2 0.999, SIFT 0.001) in heterozygous form. This missense mutation (c.7048G > A, pAla2350Thr) which confirmed by Sanger sequencing presented in Fig. 2A is one of the disease-causing mutations that has been published in previous studies. Fig. 2B showed that the mutation p.Ala2350Thr was located on the conservative region of *RYR1* so this substitution has affected the function of RYR1 protein.

## 3 Discussion

MH is an autosomal dominant pattern and the mutations in *RYR1* and *CACNA1S* were shown to relate to this disease [3]. In Vietnam, our study was the first and only case reported MH with cardiac surgery using inhaled anaesthetic drug during last ten years. In our study, the patient was performed whole exome sequencing and found no mutant variant of *CACNA1S* but a mutant variant of *RYR1* gene. *RYR1* gene mutation is associated with several diseases for example congenital myopathy or central core disease (CCD), heat/exercise-induced exertional rhabdomyolysis, atypical periodic paralysis and malignant hyperthermia [9,10]. So far, more than 150 point mutations have been found in *RYR1*, mainly occurred at three “hot spots” located in the N- terminal (exon 1-17), central (exon 39-46), C-terminal region (exon 90-104) [11]. Up to present, 42 missense mutations in the coding region of the *RYR1* cDNA have been found to associated with MH [12]. In our case report, one missense mutation (codon 2350, c.7048G > A, p.Ala2350Thr, polyphen2 0.999, SIFT 0.001) located in Bsol region (Fig. 2B), might affected the interaction with Ca^2+^/CaM-dependent protein kinase II (CaMKII) phosphorylation sites and has affected the function of RYR1 protein [13]. This might be reason to explain for MH development in patient triggered with both intravenous and inhaled anesthetics. From this report, we recommended that *RYR1* gene testing should be perform for patient who will be applied inhaled anesthetics like Sevofluran, especially for cardiac surgery. To optimize the efficacy of diagnostic and money saving for patient, we also recommended that only missense mutation of *RYR1* gene (c.7048G > A, pAla2350Thr) should be analyzed using Sanger sequencing. In case, the patient carried a mutant allele of *RYR1* gene, surgeon should consider using other anesthetics instead of Sevofluran.

## 4 Conclusion

Cardiac surgical patient was expressed malignant hyperthermia (MH) after cardiac surgery using both intravenous and inhaled anesthetics. Patient had no mutant in *CACNA1S* and a mutant at position c.7048G > A or p.Ala2350Thr in *RYR1* gene that might be the reason of MH development in this patient.

## Ethical approval

The study received the permission of ethical and scientific committee of E hospital to publish patient as paper.

## Sources of funding

This case study was funded as project KH05-2018 by E hospital, Vietnam.

## Author contribution

Tran-Thuy Nguyen, Ngoc-Thanh Le, The Binh Nguyen, Anh Tien Do, Cong Huu Nguyen, Trung-Hieu Nguyen belonged to patient's management and clinical data collection team; Nguyen Huy Hoang, Nguyen Thi Kim Lien, Dinh Doan Long performed whole exome analysis; Nguyen Thi Thuy Mau, Pham Thi Hong Nhung input and processed the patient's data; Nguyen Thi Thuy Mau and Tran-Thuy Nguyen did the draft writing; Vu Thi Thom took care of designing the experiment, revising and submitting the manuscript. All authors read and approved final manuscript.

## Research registry number

As this is a case report and not a human studies, it is exempt from registering.

## Guarantor

Le Ngoc Thanh, PhD, MD; Director of E hospital and Dean of School of Medicine and Pharmacy.

## Consent

Written informed consent was obtain from the patient for publication of this case report and accompanying images. A copy of the written consent is available for review by the Editor-in-Chief of this journal upon request.

## Provenance and peer review

Not commissioned, externally peer-reviewed.

## Declaration of competing interest

No conflicts of interest.
